# Stabilization of the catalytically active structure of a molybdenum‐dependent formate dehydrogenase depends on a highly conserved lysine residue

**DOI:** 10.1111/febs.70048

**Published:** 2025-03-03

**Authors:** Feilong Li, Michael Lienemann

**Affiliations:** ^1^ Department of Bioproducts and Biosystems Aalto University Espoo Finland; ^2^ VTT Technical Research Centre of Finland Ltd. Espoo Finland

**Keywords:** CO_2_ utilization, lysine, molybdenum‐dependent formate dehydrogenase, redox cofactor, site‐directed mutagenesis

## Abstract

Molybdenum‐dependent formate dehydrogenases (Mo‐FDHs) reversibly catalyze the interconversion of CO_2_ and formate, and therefore may be utilized for the development of innovative energy storage and CO_2_ utilization concepts. Mo‐FDHs contain a highly conserved lysine residue in the vicinity of a catalytically active molybdenum (Mo) cofactor and an electron‐transferring [4Fe‐4S] cluster. In order to elucidate the function of the conserved lysine, we substituted the residue Lys44 of *Escherichia coli* formate dehydrogenase H (*Ec*FDH‐H) with structurally and chemically diverse amino acids. Enzyme kinetic analysis of the purified *Ec*FDH‐H variants revealed the Lys‐to‐Arg substitution as the only amino acid exchange that retained formate oxidation catalytic activity, amounting to 7.1% of the wild‐type level. Ultraviolet–visible (UV–Vis) spectroscopic analysis indicated that >90% of the [4Fe‐4S] cluster was lost in the case of *Ec*FDH‐H variants ‐K44E and ‐K44M, whereas the cluster occupancy of the K44R variant decreased by merely 4.5%. Furthermore, the K44R substitution resulted in a slight decrease in its melting temperature and a significant formate affinity decrease, apparent as a 32‐fold *K*_m_ value increase. Consistent with these findings, molecular dynamics simulations predicted an increase in the backbone and cofactor mobility as a result of the K44R substitution. These results are consistent with the conserved lysine being essential for stabilizing the catalytically active structures in *Ec*FDH‐H and may support engineering efforts on Mo‐FDHs to design more efficient biocatalysts for CO_2_ reduction.

## Graphical abstract

Molybdenum‐dependent formate dehydrogenases (Mo‐FDHs) contain a strictly conserved lysine residue (K44) in the vicinity of an electron‐transferring [4Fe‐4S] cluster and a catalytically active molybdenum atom coordinated by two molybdopterins (MPT‐1 and MPT‐2). By kinetic and spectroscopic characterizations and molecular dynamics simulations of the K44 substitution variants, we demonstrate the role of this residue in stabilizing the catalytically active structures in *Escherichia coli* formate dehydrogenase H. The results of this study support engineering efforts of Mo‐FDHs to design more efficient biocatalysts for CO_2_ reduction. This figure was prepared using the ChimeraX protein structure visualization and analysis tool (version 1.8).

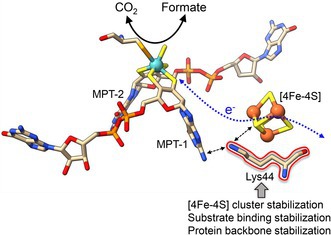

**Abbreviations**
Amp: ampicillin
BV: benzyl viologen
CD: circular dichroism
Chl: chloramphenicol
CO: cholesterol oxidase
CV: column volume
DMSOR: dimethyl sulfoxide reductase
DOPE: discrete optimized protein energy
DTT: dithiothreitol
*Ec*FDH‐H: *Escherichia coli* formate dehydrogenase H
ET: electron transfer
FAD: flavin adenine dinucleotide
Hyd: hydrogenase
IPTG: isopropyl β‐D‐1‐thiogalactopyranoside
LB: lysogeny broth
MD: molecular dynamics
MES: 2‐(N‐morpholino)ethanesulfonic acid
Mo(MGD)_2_
: Mo‐bis‐pyranopterin guanine dinucleotide
MPT: molybdopterin
NAR: nitrate reductase
OD_600_
: optical density at *λ* = 600 nm
PDB: Protein Data Bank
RMSD: root mean square deviation
SDS/PAGE: sodium dodecyl sulfate/polyacrylamide gel electrophoresis
Sec: selenocysteine
SPC: simple point charge
UV–Vis: ultraviolet–visible
WT: wild‐type

## Introduction

Nearly 100 different reactions are catalyzed by molybdenum enzymes and include two‐electron redox reactions essential for nitrogen, sulfur, and carbon metabolism cycles in all kingdoms of life [1, 2, 3]. A common feature of these enzymes is a mononuclear Mo cofactor that, with the exception of the rare multi‐nuclear [MoFe_7_S_9_] cluster of nitrogenases [2, 3, 4, 5, 6], is coordinated by a dithiolate group of a pterin derivative. Mononuclear Mo‐enzymes can be divided into three families with highly diverse structures and catalytic functions, exemplified by enzymes xanthine oxidase, sulfite oxidase, and dimethyl sulfoxide reductase (DMSOR) [2, 7] of which the latter is the largest. The Mo atom of members of the DMSOR family is coordinated by two identical pterin derivatives (MPT‐1 and MPT‐2), forming a Mo‐bis‐pyranopterin guanine dinucleotide (Mo(MGD)_2_) cofactor [2, 8, 9]. As an important subgroup in the DMSOR family, Mo‐dependent formate dehydrogenases (Mo‐FDHs) are capable of rapidly catalyzing the reversible CO_2_–formate interconversion and are involved in various biological pathways in the biosynthetic and energy metabolism [8, 10, 11]. This reaction is of great interest in biotechnological applications because it allows CO_2_ sequestration as the useful commodity formate that has been strongly advocated as an energy storage chemical for the hydrogen economy [12, 13, 14].

**Fig. 1:**
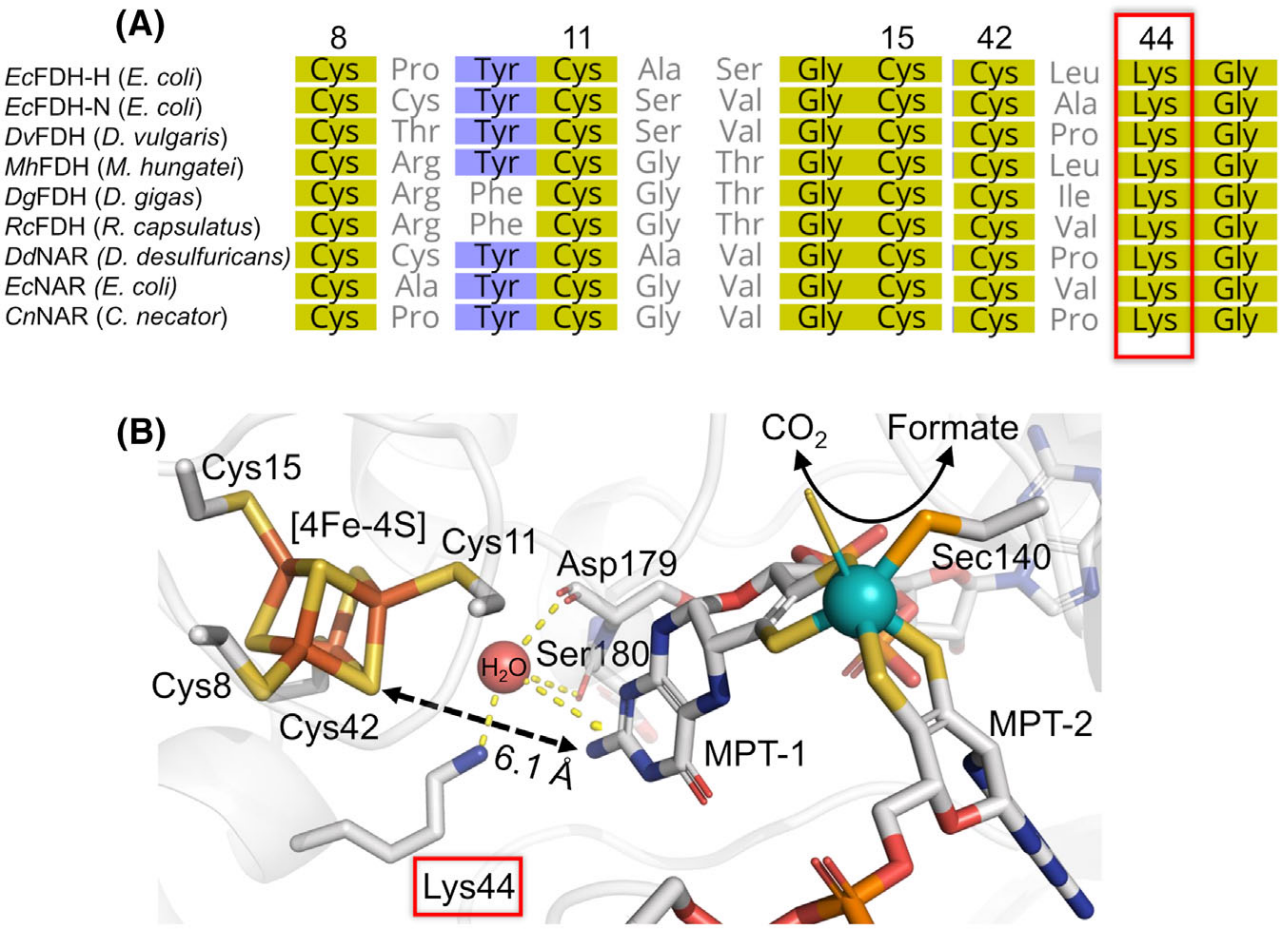
Sequence homology analysis and active‐site structure of the Mo‐dependent *E. coli* dehydrogenase H (*Ec*FDH‐H). (A) Amino acid sequence alignment of *Ec*FDH‐H and its homologous enzymes, including five molybdenum (Mo)/tungsten (W)‐dependent formate dehydrogenases [PDB identification codes: 1KQF (*Ec*FDH‐N), 6SDR (*Dv*FDH), 7BKB (*Mh*FDH), 1H0H (*Dg*FDH), and 6TG9 (*Rc*FDH)] as well as three nitrate reductases (NARs) [PDB IDs: 2JIO (*Dd*NAR), 2NYA (*Ec*NAR), and 3O5A (*Cn*NAR)] were performed using the Geneious Prime software tool (Biomatters; Auckland, New Zealand). The conserved [4Fe‐4S] cluster‐coordinated cysteine residues are numbered according to the amino acid sequence of *Ec*FDH‐H. The lysine residue investigated in this study is highlighted in a red box, and the varying degrees of homology are indicated by different colors (yellow > blue). (B) Active site structure of *Ec*FDH‐H (PDB ID: 1FDO) visualized using the PyMOL Molecular Graphics System (Version 1.7.2, Schrödinger; New York City, NY, USA). The enzyme‐bound [4Fe‐4S] cluster, amino acid residues, and the molybdopterin groups (MPT‐1 and MPT‐2) in *Ec*FDH‐H are shown in stick representation, except the Mo ion and the water molecule shown in spheres. Yellow dotted lines between the [4Fe‐4S] cluster, residues Lys44, Asp179, and Ser180, a local water molecule and the MPT‐1 group indicate hydrogen bonding. The identity of iron, sulfur, carbon, nitrogen, oxygen, and molybdenum atoms is indicated by coloring in brown, yellow, gray, blue, red, and cyan, respectively.

Mo‐FDHs exhibit considerable diversities in their subunit arrangement and cofactor makeup but the Mo(MGD)_2_ cofactor and its adjacent [4Fe‐4S] cluster are ubiquitous groups across all of these enzymes [15]. This [4Fe‐4S] cluster constitutes the first electron transfer (ET) relay to the catalytically active Mo ion in the catalytic subunit of Mo‐FDHs and the inter‐cofactor ET may be a critical step governing their catalytic activity [2, 15, 16, 17]. Noticeably, a strictly conserved lysine residue was found between the two redox cofactors [18, 19, 20] (Fig. 1A) and was initially proposed to directly mediate the inter‐cofactor ET [21]. In view of the 14‐Å rule of physiologically relevant electron tunneling in proteins [22, 23], the short distance between these two cofactors, e.g., an edge‐to‐edge distance of 6.1 Å in the Mo‐dependent FDH‐H from *E. coli* (*Ec*FDH‐H; Fig. 1B), may support a direct inter‐cofactor ET instead of the lysine‐mediated mechanism [21]. It is worth noting that the lysine residue at equivalent sites of other DMSOR family enzymes, such as nitrate reductases (NARs) and DMSORs, has been proposed to fine‐tune the reduction potential of its neighboring [4Fe‐4S] cluster for reducing ET barriers [24, 25, 26]. Nevertheless, due to different catalytic functions of DMSORs, NARs, and Mo‐FDHs [8], further experimental evidence is needed to elucidate the function of the conserved lysine in the catalytic mechanism of Mo‐FDHs.

In this study, we examined the role of the *Ec*FDH‐H residue K44 in the catalysis of CO_2_–formate interconversion by introduction of structurally and chemically diverse amino acid substituents. The K44‐substitution variants of *Ec*FDH‐H were compared with the wild‐type (WT) enzyme based on kinetic and spectroscopic analysis as well as molecular dynamics simulations to gain an understanding of the molecular mechanisms in which the K44 residue participates and contributes to the catalytic function of *Ec*FDH‐H.

## Results

### The K44R substitution led to partial inactivation of *Ec*FDH‐H

**Fig. 2:**
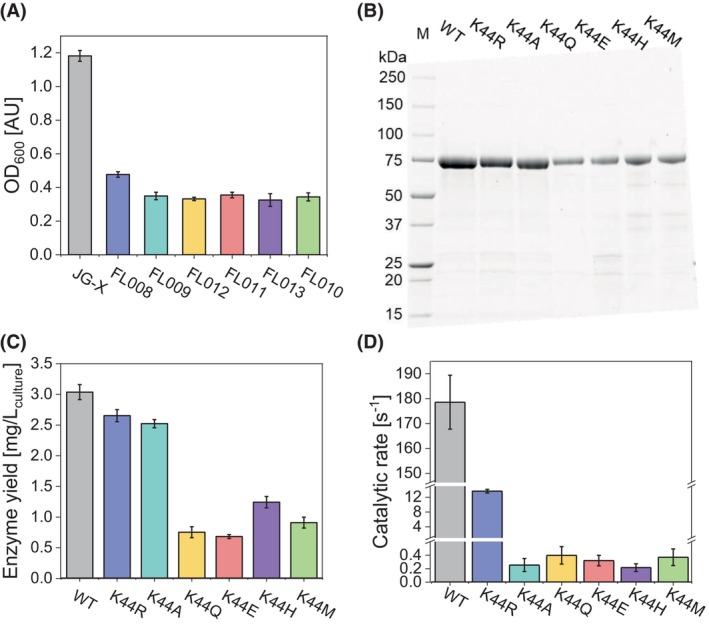
Analyses of *Ec*FDH‐H [wildtype (WT)] and its variants ‐K44R, ‐K44A, ‐K44M, ‐K44E, ‐K44Q, and ‐K44H as well as their *E. coli* production host strains JG‐X, FL008, FL009, FL010, FL011, FL012, and FL013, respectively. (A) Final optical density at *λ* = 600 nm (OD_600_) of *E. coli* strains after anaerobic cell cultivation in gas‐tight 2‐L laboratory bottles containing 1 L lysogeny broth (LB) medium supplemented with 4 g·L^−1^ glucose, 1 mm Na_2_MoO_4_ and 10 μm Na_2_SeO_3_. (B) Sodium dodecyl sulfate/polyacrylamide gel electrophoresis (SDS/PAGE) analysis of reference proteins (lane labeled M) and purified *Ec*FDH‐H (WT) as well as its K44 substitution variants using 4–20% acrylamide gradient. (C) Enzyme yields obtained from lysed *E. coli* cells by Ni‐ion affinity chromatography under strictly anaerobic conditions. (D) Formate oxidation rates determined by spectrometry at *λ* = 555 nm and 25 °C under strictly anaerobic conditions in 50 mm phosphate buffer (pH 7.5) containing the purified enzymes at 1.5 μg·mL^−1^ [*Ec*FDH‐H (WT)], 7.5 μg·mL^−1^ (‐K44R), and 30 μg·mL^−1^ (‐K44A, ‐K44E, ‐K44Q, ‐K44M, and ‐K44H) as well as 10 mm formate and 2 mm oxidized benzyl viologen (BV^2+^). Data are mean values of three independent experiments and the error bars indicate the standard deviation.

To study the function of the conserved Lys44 in catalysis of *Ec*FDH‐H, we replaced this residue with six amino acids representing a wide range of steric and chemical properties. These included the bulkier and alkaline arginine, hydrophobic methionine, small and chemically inert alanine, imidazole‐bearing histidine and anionic glutamic acid as well as its uncharged structural homolog glutamine. *Ec*FDH‐H‐K44 substitution variants bearing a C‐terminal His_6_‐tag were produced anoxically using derivatives of *E. coli* strain FL003 harboring suitable expression vectors (Table 1). As apparent from a reduction in the final cell density (OD_600_) measured following fermentative cultivation with respect to the WT‐producing *E. coli* strain JG‐X (final OD_600_ = 1.18; Fig. 2A), the performed amino acid substitutions resulted in a strong growth inhibition of *E. coli* strains producing these variants (final OD_600_ = 0.33–0.48). While none of the K44 substitutions led to *Ec*FDH‐H truncation (Fig. 2B), a clear decrease in enzyme yields from the WT level of 3.04 ± 0.12 mg·L_culture_^−1^ to <0.68 ± 0.03 mg·L_culture_^−1^ was observed (Fig. 2C). Next, the catalytic activity of *Ec*FDH‐H (WT) and the purified K44 substitution variants was analyzed by monitoring the production of BV^+^ ions in the presence of the *Ec*FDH‐H substrates formate (*c*_final_ = 10 mm) and oxidized benzyl viologen (BV^2+^) (*c*_final_ = 2 mm). The catalytic rate of the WT enzyme was determined as 177.0 ± 10.7 s^−1^, which agrees well with the conversion rate of 160 s^−1^ that was reported earlier by Bassegoda *et al*. and measured at similar conditions (10 mm formate and 1 mm BV^2+^) [27]. Both enzymatic rates are lower than the maximum turnover rate (*k*_cat_) of *Ec*FDH‐H (1415 s^−1^) reported by Axley *et al*. [28] which is due to the fact that the activity measurements reported here and by Bassegoda *et al*. were performed at nonsaturating substrate concentrations. Notably, the determination of *k*_cat_ for enzymes catalyzing two‐substrate reaction requires an extrapolation of measured data which may result in a large deviation and is therefore not considered herein as well as other recent works on Mo‐FDHs [27, 29]. An almost complete enzyme inactivation was observed for variants ‐K44A, ‐K44M, ‐K44E, ‐K44Q, and ‐K44H retaining <0.23% activity of the activity of the WT enzyme while the K44R variant retained a low residual activity of 12.54 ± 0.19 s^−1^, corresponding to ~7.08% of the WT level (Fig. 2D).

**Table 1:** *Escherichia coli* strain JG‐X (MC4100 *ΔfdhF*, *ΔiscR*) and its derivatives used for producing *Ec*FDH‐H (WT) and its K44 variants^b^.

Strains	Plasmid‐encoded FDHs	Plasmids	VTT‐CC ID
JG‐X^a^	*Ec*FDH‐H	pSU21‐*selC*, Trc99a‐*fdhF*	E‐223614
FL003	–	pSU21‐*selC*	E‐233617
FL008	*Ec*FDH‐H‐K44R	pSU21‐*selC*, pFL006	E‐233622
FL009	*Ec*FDH‐H‐K44A	pSU21‐*selC*, pFL007	E‐233623
FL010	*Ec*FDH‐H‐K44M	pSU21‐*selC*, pFL008	E‐243627
FL011	*Ec*FDH‐H‐K44E	pSU21‐*selC*, pFL009	E‐243628
FL012	*Ec*FDH‐H‐K44Q	pSU21‐*selC*, pFL010	E‐243629
FL013	*Ec*FDH‐H‐K44H	pSU21‐*selC*, pFL011	E‐243630

^a^ Source: Shelley D. Minteer (Missouri University of Science and Technology, Rolla, MO, USA) [50].

^b^ VTT Culture Collection strain identifiers (VTT‐CC IDs) are indicated.

### K44 substitutions destabilized the binding of the [4Fe‐4S] cluster in *Ec*FDH‐H

**Fig. 3:**
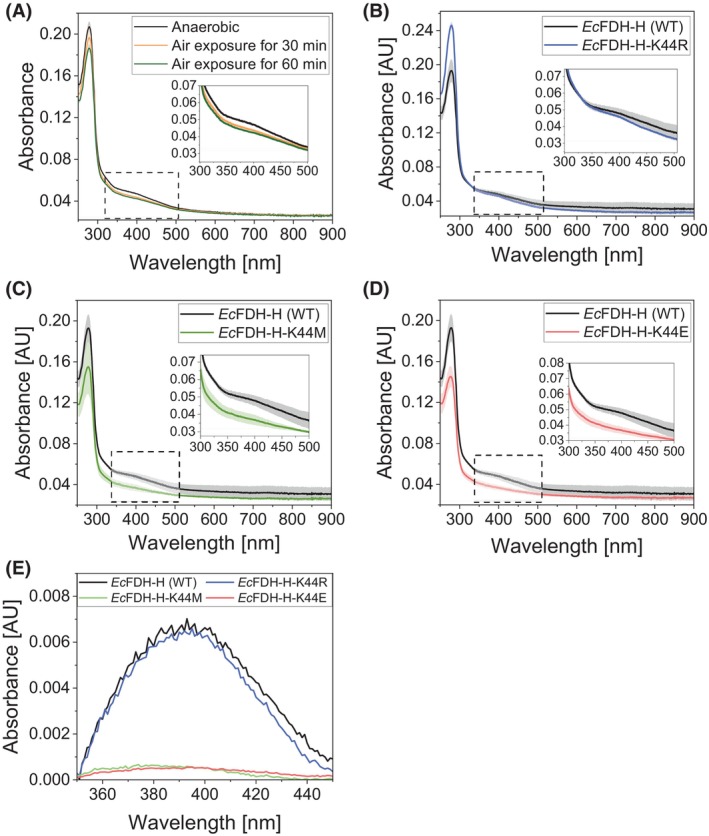
Ultraviolet–visible (UV–Vis) spectroscopic analysis of *Ec*FDH‐H [wildtype (WT)] and its variants ‐K44R, ‐K44M, and ‐K44E. (A) UV–Vis absorption spectra of *Ec*FDH‐H (WT) dissolved at a concentration of 2.5 mg·mL^−1^ in 25 mm 2‐(N‐morpholino)ethanesulfonic acid (MES) buffer (pH 6.0) containing 100 mm Na_2_SO_4_. The data sets were recorded using freshly purified samples (anaerobic) and samples exposed to air for 30 min and 60 min at 4 °C and 1200 rpm orbital shaking. Comparison of the UV–Vis spectra of the freshly purified enzyme solutions containing *Ec*FDH‐H (WT) and its variants K44R, ‐K44M, and ‐K44E (B–D, respectively) at a concentration of 2–3 mg·mL^−1^. The enlarged regions containing spectroscopic data at *λ* = 300–500 nm are shown as insets and the shaded areas indicate the standard deviation of data measured from three independent experiments (*n* = 3). (E) Spectral signals at *λ* = 350–450 nm were obtained by baseline subtraction from the data shown in B–D.

Intrigued by the spatial proximity of Lys44 to the single [4Fe‐4S] cluster in the structure of *Ec*FDH‐H [21], we probed the effect of K44 substitutions on the occupancy of the [4Fe‐4S] cluster by comparison of the ultraviolet–visible (UV–Vis) spectra of *Ec*FDH‐H (WT) and its variants ‐K44R, ‐K44M, and ‐K44E. As shown in Fig. 3A, a wide characteristic ‘shoulder’ around at *λ* = 400 nm can be discerned for *Ec*FDH‐H (WT) under strictly anaerobic conditions and is indicative of the presence of an oxidized [4Fe‐4S] cluster [30, 31]. This absorbance signal assignment is consistent with previously reported UV–Vis spectroscopic analyses of the *Desulfovibrio vulgaris* tungsten‐dependent formate dehydrogenase *Dv*FDH [32], the *Chlamydomonas reinhardtii* [FeFe] hydrogenase HydA1 [33], and the marine *Methanococcales* thermophilic nitrogenase reductases *Av*NifH, *Mi*NifH, *Mm*NifH, and *Mt*NifH [34]. The absorbance signal at around 400 nm disappears when the [4Fe‐4S]^2+^ cluster is oxidized to [4Fe‐4S]^3+^ upon exposure to oxygen and further decomposed to the [2Fe‐2S]^2+^ cluster that has a characteristic absorbance signal around 450 nm [35, 36]. Notably, exposure of the enzyme to air resulted in a gradual reduction in this signal. Substitution of the lysine at site 44 with the alkaline residue Arg partially retained the shoulder signal at ~400 nm while this absorbance signal was absent in the spectra of *Ec*FDH‐H variants ‐K44E and ‐K44M (Fig. 3B–D). Peak analysis performed for the absorbance spectra of the studied enzymes revealed 400‐nm signal peak height reductions of the *Ec*FDH‐H variants from 6.6 × 10^−3^ (WT) to 6.3 × 10^−3^ (‐K44R), 0.50 × 10^−3^ (‐K44E) and 0.52 × 10^−3^ (‐K44M) (Fig. 3E).

### The K44R substitution significantly reduced the binding affinity of *Ec*FDH‐H for its substrate formate

**Fig. 4:**
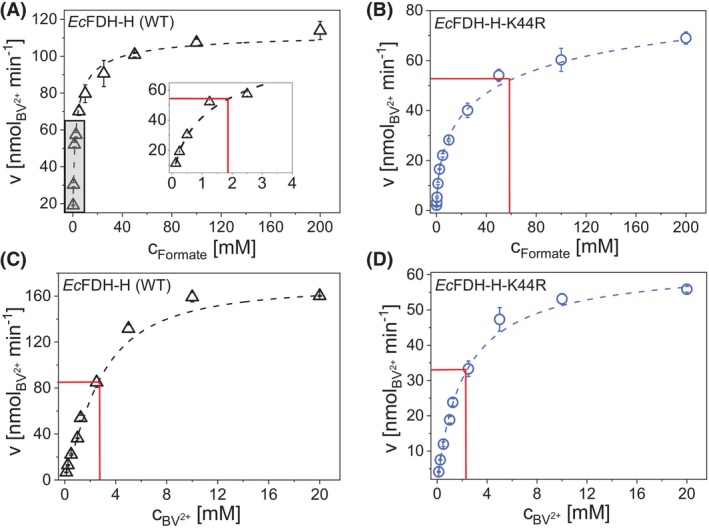
Kinetic analysis of *Ec*FDH‐H [wildtype (WT)] and its variant K44R. Effect of formate (A and B) or oxidized benzyl viologen (BV^2+^) (C and D) concentration on the activity of *Ec*FDH‐H (WT) (black) and the K44R variant (light blue). The 200‐μL reaction mixture contains the tested *Ec*FDH‐H (WT) and K44R enzyme samples at a concentration of 1.5 and 7.5 μg·mL^−1^, respectively, as well as, the respective co‐substrates at BV^2+^ [2 mm] and formate [10 mm]. The consumption of BV^2+^ was monitored for 60 s at 25 °C under strictly anaerobic conditions. The inset of Panel A displays the activity data recorded for *Ec*FDH‐H (WT) at formate concentrations ranging between 0 and 4 mm (shaded area). Estimation of the kinetic parameters $K_{m}^{\text{Formate}}$ and $K_{m}^{{\mathrm{BV}}^{2+}}$ were performed by fitting the data to the Michaelis–Menten equation (dashed line), in which red lines intersect the y‐axis at the apparent ½ *V*_max_ and indicate the respective *K*_m_ values as x‐axis intersections. Data are mean values of three independent experiments with error bars indicating the standard deviation.

**Fig. 5:**
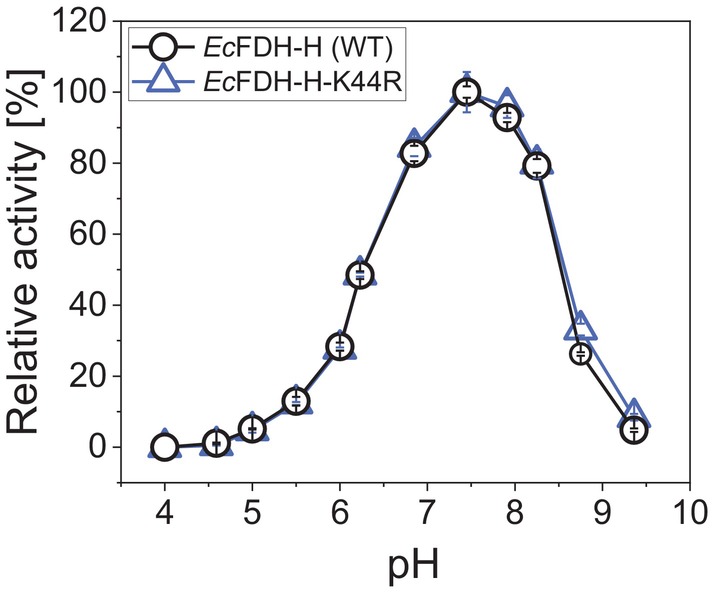
The pH dependence of the catalytic activity of *Ec*FDH‐H [wildtype (WT)] and its variant K44R. Formate oxidation activity of *Ec*FDH‐H (WT) and variant K44R measured at pH 4.0–9.5 in 50 mm buffer (pH 3.0–6.0: citrate; pH 6.0–9.5: bis‐tris propane) prepared by addition of aqueous NaOH and H_2_SO_4_ solutions to avoid *Ec*FDH‐H inhibition by chloride ions [55]. The displayed relative activity is determined by normalizing the measured activity at the respective pH to the maximum activity at pH 7.5. The formate oxidation activity of *Ec*FDH‐H enzymes was quantified spectroscopically at *λ* = 555 nm inside an anaerobic cabinet. The reaction mixture contained the reaction substrates formate [10 mm] and oxidized benzyl viologen (BV^2+^) [2 mm] as well as either 1.5 μg·mL^−1^ *Ec*FDH‐H (WT) or 7.5 μg·mL^−1^ *Ec*FDH‐H‐K44R. The measurements were performed in triplicate and error bars indicate the standard deviation.

Comparative kinetic analysis of the K44R variant and WT enzyme revealed a significantly decreased binding affinity for formate when compared to the WT enzyme as evident from the respective calculated Michaelis–Menten constants ($K_{m}^{\text{formate}})$ of 1.85 ± 0.27 mm and 58.61 ± 6.15 mm (Fig. 4A,B). The affinity of the K44R variant for the electron acceptor BV^2+^, on the contrary, was found to be slightly increased as apparent from $K_{m}^{\mathrm{BV}2+}$ values of 2.21 ± 0.37 mm and 2.75 ± 0.50 mm determined for the K44R variant and *Ec*FDH‐H (WT), respectively (Fig. 4C,D). Additional formate oxidation measurements performed with both enzymes at pH 3.0–9.5 revealed highly similar activity profiles with a pronounced maximum at pH 7.5 (Fig. 5).

### The K44R substitution decreased the thermal stability of *Ec*FDH‐H

**Fig. 6:**
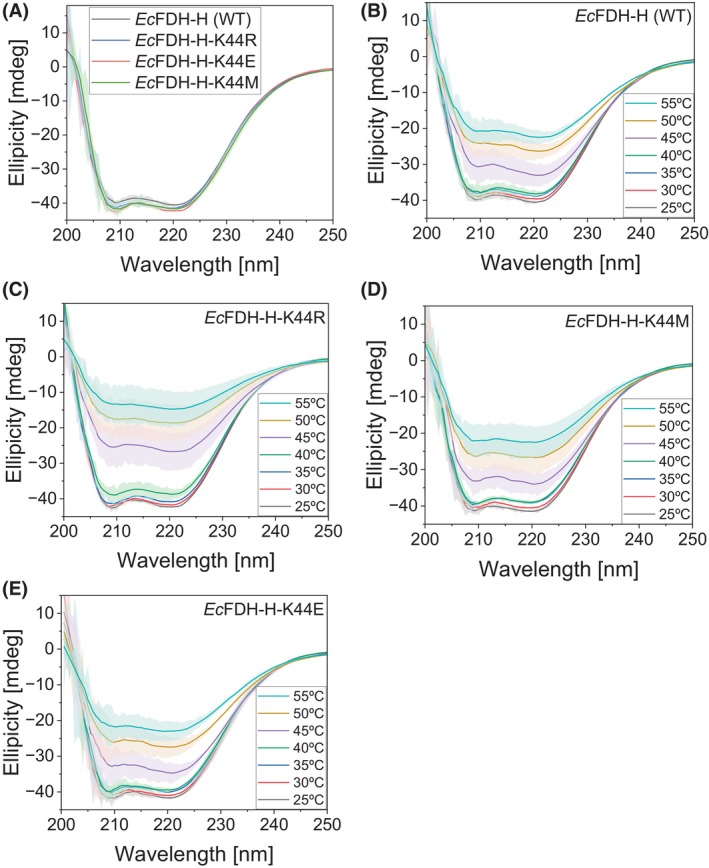
Circular dichroism (CD) analysis of *Ec*FDH‐H [wildtype (WT)] and its variants ‐K44R, ‐K44M, and ‐K44E. The CD ellipticity was measured at 25 °C (A) and at temperatures varying between 25 and 55 °C (B–E). Signals were recorded from 300 μL samples containing 100 mm Na_2_SO_4_ and ~0.5 mg·mL^−1^ enzyme dissolved in 25 mm 2‐(N‐morpholino)ethanesulfonic acid (MES) buffer (pH 6.0). Samples were equilibrated for 60 s at the respective temperature in sealed QS‐110 quartz cuvettes prior to CD signal recording. Data are mean values of three independent experiments with error bars indicating the standard deviation.

**Fig. 7:**
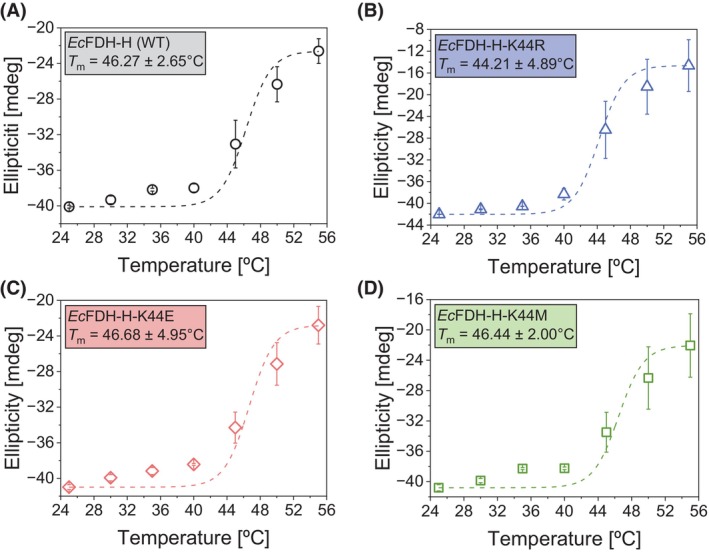
Thermal stability analysis of *Ec*FDH‐H [wildtype (WT)] and its variants ‐K44R, ‐K44E and ‐K44M. Melting curves of *Ec*FDH‐H (WT) (A) and ‐K44R (B), ‐K44E (C) and ‐K44M (D) obtained by nonlinear least squares fitting of circular dichroism (CD) signals recorded at *λ* = 222 nm and different temperatures (Fig. 6) to the Boltzmann equation using *T*_m_ as midpoint value x_0_ [56]. The displayed data are mean values calculated from three biological replicates (*n* = 3) with error bars indicating the standard deviation.

Next, circular dichroism (CD) spectroscopy was used to investigate structural changes caused by the substitution of residue K44 with Arg, Glu, and Met. CD spectra were recorded at *λ* = 200–250 nm to minimize signal noise due to strong light absorption of 2‐(N‐morpholino)ethanesulfonic acid (MES) buffer at *λ* <200 nm. As shown in Fig. 6, the CD spectra of all tested *Ec*FDH‐H enzymes were indistinguishable from each other at *T* = 25–35 °C. In the case of variant ‐K44R, a slight shift toward less negative ellipticity values could be discerned at *T* = 40 °C across the monitored *λ* range while the spectra of *Ec*FDH‐H (WT), ‐K44M, and ‐K44E closely resembled those recorded at lower temperatures. Upon further increasing temperature to 55 °C, the signal intensity of all enzyme samples was significantly reduced and among the tested the *Ec*FDH‐H enzymes the strongest ellipticity signal attenuation was observed for the K44R variant. To quantify these CD signal changes, the melting temperature (*T*_m_) of each enzyme was determined based on the ellipticity change at 222 nm. For the *Ec*FDH‐H variant ‐K44R, the *T*_m_ amounted to 44.21 ± 4.89 °C, which was slightly lower than the values calculated for *Ec*FDH‐H (WT) (46.27 ± 2.65 °C), ‐K44E (46.68 ± 4.95 °C) and ‐K44M (46.44 ± 2.00 °C) (Fig. 7).

### MD simulation reveals a destabilization of Mo(MGD)_2_ cofactor binding resulting from the substitution of *Ec*FDH‐H residue Lys44 with Arg

**Fig. 8:**
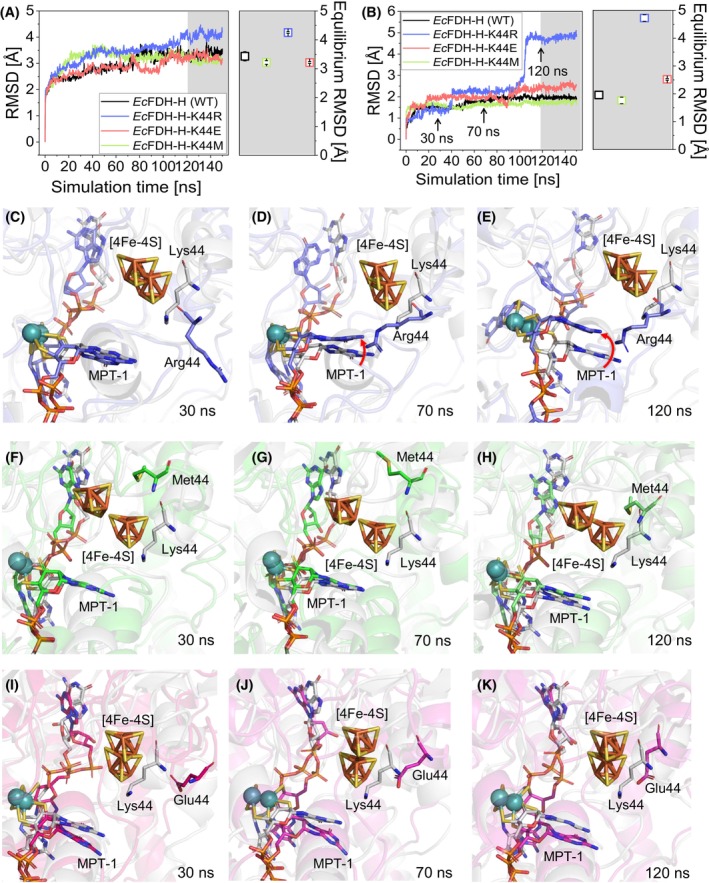
Molecular dynamics (MD) simulation of *Ec*FDH‐H [wildtype (WT)] and its variants ‐K44R, ‐K44M, and ‐K44E. The system was simulated at a temperature of 45 °C using the Desmond MD engine (D. E. Shaw Research; New York City, NY, USA). The root mean square deviation (RMSD) of protein backbone‐C_α_ atoms (A) and the bound Mo‐bis‐pyranopterin guanine dinucleotide (Mo(MGD)_2_) cofactor (B) throughout the 150‐ns simulation period are displayed. Each enzyme variant is represented by a single RMSD data set (*n* = 1). Visualization of the active‐site structures of the *Ec*FDH‐H variants ‐K44R (C–E), ‐K44M (F–H), ‐K44E (I–K) and *Ec*FDH‐H (WT) was performed using the PyMOL Molecular Graphics System (Version 1.7.2, Schrödinger; New York City, NY, USA). The displacement of the molybdopterin group of Mo(MGD)_2_ cofactor proximal to site 44 in *Ec*FDH‐H (MPT‐1) in the ‐K44R variant is marked with red arrows.

To investigate the reasons for the differences of catalytic and structural properties between *Ec*FDH‐H (WT) and K44 variants, the stability of the protein backbone and the Mo(MGD)_2_ cofactor of *Ec*FDH‐H (WT), ‐K44E, ‐K44M, and ‐K44R were estimated using MD simulations. During a 120–150 ns equilibrium period, *Ec*FDH‐H (WT), ‐K44E, and ‐K44M were highly similar as judged from the root mean square deviation (RMSD) of the position of the backbone‐C_α_ being in the range of 3.20–3.43 Å. In the case of *Ec*FDH‐H‐K44R, a gradual increase in the backbone‐C_α_ movement from 3.5 Å was predicted to occur after the 100‐ns timepoint that reached an 4.30 Å average value during *t* = 120–150 ns (Fig. 8A) and coincides with an abrupt RMSD doubling at 100 ns predicted for its Mo cofactor (Fig. 8B). All simulated Mo movements remained at largely constant levels after the 100‐ns timepoint but differed in their average equilibrium RMSD from the one of the K44R variant being 4.72 Å and significantly larger than that of *Ec*FDH‐H (WT) (1.95 Å), *Ec*FDH‐H‐K44E (1.76 Å) and *Ec*FDH‐H‐K44M (2.52 Å).

In order to identify the *Ec*FDH‐H regions that are affected by K44 substitutions and undergo concerted motions with the backbone‐C_α_ atoms and the Mo(MGD)_2_ cofactor, the structures of *Ec*FDH‐H (WT) and the K44 substitution variants, predicted at timepoints 30, 70, and 120 ns were compared. When comparing the *Ec*FDH‐H (WT) and the K44R variant, the structural similarity of their Mo‐bound MPT‐1 groups at 30 ns is notable as, at this timepoint, the backbone‐C_α_ atoms of residues R44 and K44 were predicted to be located 5.1 Å apart and the positions of the [4Fe‐4S] clusters of these enzymes differed by 2.3 Å. In the case of variant K44R, a gradual shift of the Mo atom and its complexing MPT‐1 group away from position 44 is predicted between timepoints 30 and 120 ns in addition to a 5.5‐Å movement of the MPT‐1 amine. Moreover, the simulation results indicate that the [4Fe‐4S] clusters of both enzymes remain at a constant distance to each other during the simulated timeframe. On the contrary, no significant movement of these groups is predicted to occur in the case of the WT enzyme (Fig. 8C–E). In the case of the *Ec*FDH‐H variants ‐K44E and ‐K44M, the [4Fe‐4S] clusters were located at different positions at the 30 ns timepoint at a distance of 2.8 and 5.1 Å to the [4Fe‐4S] cluster of the WT, respectively, and did not largely change their position until 120 ns. The conformational changes in the Mo(MGD)_2_ cofactor were comparably small involving a 1.9 and 0.7 Å relocation of the amine nitrogen atom of the MPT‐1 group from its original position at the 30 to 120 ns timepoints, respectively (Fig. 8F–K).

## Discussion

In order to increase the understanding of the roles of conserved amino acid residues in the catalysis of Mo‐FDHs, we substituted the strictly conserved *Ec*FDH‐H residue K44 with structurally and chemically diverse amino acids. Based on the high conservation of the K44 residue and its location between the electron‐transferring Mo(MGD)_2_ cofactor and the [4Fe‐4S] cluster as well as the recently demonstrated function of *Ec*FDH‐H in supporting the fermentative growth of *E. coli* by the remediation of formate accumulation and cytosol acidification [37], a negative effect of *Ec*FDH‐H‐K44 substitutions on the *E. coli* fitness during fermentative growth was expected and confirmed by anaerobic cultivation trials during which significant growth inhibition of *E. coli* strains producing *Ec*FDH‐H‐K44 variants was observed (Fig. 2A). Among the assayed strains, FL008 stood out as its growth was least inhibited and its produced *Ec*FDH‐H‐K44R retained ~7.08% of the activity of the WT enzyme. This observation may be taken as an indication for the dependence of the catalytic mechanism on a positive charge at position 44 that is provided by Lys and Arg at physiological conditions. This is supported by the highly similar basicity of K44 and R44 in *Ec*FDH‐H (p*K*_a_ of 11.3 and 12.3, respectively; predicted with the Prop*K*a online tool [38]), the highly similar activity profiles of *Ec*FDH‐H (WT) and ‐K44R at pH 4.0–9.5 and previous studies demonstrating the existence of cationic arginine residues in different protein micro‐environments [39].

Support for a stabilizing effect of Lys44 on its adjacent [4Fe‐4S] cluster was obtained by UV–Vis spectroscopic analysis revealing the absence of a characteristic [4Fe‐4S]^2+^ signal at ~400 nm [40] in the absorbance spectra of *Ec*FDH‐H variants ‐K44E and ‐K44M and its partial preservation in the K44R variant (Fig. 3). The partially retained [4Fe‐4S] cluster occupancy and catalytic activity of the K44R variant indicate that the cationic side chain at site 44 stabilizes the [4Fe‐4S] cluster and thereby facilitates intramolecular ET in *Ec*FDH‐H. This idea is in accordance with a previous study reporting a significantly reduced iron–sulfur cluster occupancy of the NAR of *Synechococcus* sp. PCC 7942 harboring an Arg‐to‐Glu substitution at position 58 that corresponds to the position 44 in *Ec*FDH‐H [41]. Also, Maslać *et al*. found positively charged patches around the [4Fe‐4S] clusters of the marine *Methanococcales* nitrogenase reductases *Av*NifH, *Mi*NifH, *Mm*NifH, and *Mt*NifH, which supports the significance of proximal positively charged amino acid residues for [4Fe‐4S]‐cluster‐mediated ET [34]. Notably, further support for the importance of alkaline residues in the proximity of the iron–sulfur cluster is provided by Lys‐to‐Arg substitution trials with the *Rhodobacter sphaeroides* nitrate reductase NapAB and the *E. coli* DMSO reductase DmsABC where substitutions at the sites corresponding to K44 in *Ec*FDH‐H decreased the reduction potential of the iron–sulfur cluster and was suggested to reduce its ET capabilities via prevention of external electron uptake [24, 25, 42]. In our study, the side chain of Arg is slightly longer than that of Lys and may therefore occupy space that is required for coordination of the [4Fe‐4S] cluster by the C42 residue and thereby weaken the cluster binding in the K44R variant.

Steady‐state kinetic analysis of *Ec*FDH‐H (WT) and the K44R variant revealed a significantly decreased formate binding affinity of the K44R variant indicated by its 31‐fold increased $K_{m}^{\text{formate}}$ with respect to the WT enzyme and may be explained by the proximity of the substituted Lys to the MPT‐1 group of the Mo(MGD)_2_ cofactor that has been reported to bind formate [43]. Further investigation of the K44 substitution employed CD spectroscopy to reveal alterations of the secondary structure and thermal stability of the *Ec*FDH‐H enzyme. The high resemblance of the spectra of *Ec*FDH‐H variants ‐K44R, ‐K44E, and ‐K44M with that of *Ec*FDH‐H (WT) at 25 °C indicated that the performed K44 substitutions did not significantly alter the secondary structure while a compromised stability of the K44R variant was apparent from thermal melt analyses as a *T*_m_ reduction of ~2.06 °C as compared to *Ec*FDH‐H (WT). Polypeptide chain–cofactor interactions restrict the conformational flexibility of the holoenzyme [44, 45, 46, 47, 48] and, accordingly, the observed thermal instability of the K44R variant may be explained by a steric crowding effect that is due to the Lys residue replacement with a bulkier Arg that weakens the binding of the Mo(MGD)_2_ cofactor and [4Fe‐4S] cluster. In accordance with this notion, none of the K44 substitutions with smaller residues resulted in altered *T*_m_ although these substitutions may alter hydrogen bond networks around the substituted residue, including the proximal MPT‐1 group and a water molecule (Fig. 1B) [21]. Similar cofactor binding destabilization and protein melting temperature decrease have been reported by Caldinelli *et al*. as a result of an amino acid substitution introduced into the cofactor binding site of the *Brevibacterium sterolicum* flavin adenine dinucleotide (FAD)‐containing cholesterol oxidase CO [45]. In order to increase the understanding of possible structural changes resulting from the K44 substitutions into *Ec*FDH‐H, we performed MD simulations that predicted a profound protein backbone movement increase for the K44R variant (Fig. 8A). In the case of the RMSD profiles of *Ec*FDH‐H variants ‐K44E and ‐K44M, no statistically significant differences to the one of the *Ec*FDH‐H (WT) were found and thereby, these MD‐based predictions agree well with the CD data discussed above. In addition to the protein backbone movement, our MD simulations predicted Mo(MGD)_2_ cofactor displacements for all three modeled variants (Fig. 8C–K) with the largest structural deviation from WT visible in the K44R‐structure (Fig. 8C–E). Notably, the MD simulation method employed here is not able to predict the possible loss of the Mo(MGD)_2_ and [4Fe‐4S] cofactors and thereby motivate further experimental characterizations of *Ec*FDH‐H variants by methods that can monitor the Mo(V) occupancy of the active site, for example, electron paramagnetic resonance [49].

## Conclusions

The presented substitution analysis of *Ec*FDH‐H demonstrated that both, the charge and size of the lysine residue at position 44 determine the [4Fe‐4S] cluster occupancy, substrate binding, thermal stability, and foremost, catalytic activity of the enzyme. The substitution of the native K44 with Arg preserved the positive charge in the vicinity of the [4Fe‐4S] cluster and attenuated the cofactor occupancy only to a minor extent. On the contrary, the K44R substitution markedly decreased the formate binding affinity and catalytic activity of *Ec*FDH‐H. Overall, the presented results illustrate the sensitivity of Mo‐FDHs to amino acid substitutions close to the bound metal cofactors and the strong attenuating effect of steric crowding and loss of local charges in these regions on enzyme activity. Thereby, these findings may aid the design of efficient biocatalysts for CO_2_ reduction by alteration of other conserved residues surrounding the redox cofactors of Mo‐FDHs.

## Materials and methods

### Plasmids and strains

*Escherichia coli* strain JG‐X (MC4100, ∆*fdhF*, and ∆*iscR*) harboring plasmids pTrc99a‐*fdhF* (Amp^R^) and pSU21‐*selC* (Chl^R^) was originally prepared by members of the group of Prof. Golbeck (Pennsylvania State University, PA, USA) and kindly provided by Prof. Shelly Minteer (Missouri University of Science and Technology, Rolla, MO, USA) [50]. The *E. coli* strain FL003 harboring a single pSU21‐*selC* plasmid was prepared by elimination of the plasmid pTrc99a‐*fdhF* from strain JG‐X as previously described [15]. *E. coli* strains FL008–FL013 were used for the production of *E. coli* formate dehydrogenase H (*Ec*FDH‐H) variants in which the residue K44 was substituted with various amino acids and prepared by electro‐transformation of strain FL003 with one of the plasmids pFL006 – pFL011 (Table 1) using an MicroPulser Electroporator (Bio‐Rad; Hercules, CA, USA). All strains harbored the helper plasmid pSU21‐*selC* encoding Sec‐tRNA for overproduction of the Sec‐containing *Ec*FDH‐H enzymes. The plasmids pFL006–pFL011 were prepared by site‐directed mutagenesis of pTrc99a‐*fdhF* as described previously [15] using suitable primer pairs (Table 2) and *fdhF* gene mutations were confirmed by DNA sequencing (Microsynth Seqlab; Göttingen, Germany).

**Table 2:** Primers used for the site‐directed mutagenesis of *Ec*FDH‐H designed using the Primer X online tool (https://www.bioinformatics.org/primerx/index.htm) according to Wang et al. [54]. The respective melting temperatures (*T*_m_) are indicated and mutated bases are shown in red.

Primers	Nucleotide sequences (5′ to 3′ terminus)	*T*_m_ [°C]
K44R_F	G GGT ACC CTG TGT CTG CGA GGT TAT TAT GGC TGG G	76.9
K44R_R	C CCA GCC ATA ATA ACC TCG CAG ACA CAG GGT ACC C	76.9
K44A_F	G GGT ACC CTG TGT CTG GCA GGT TAT TAT GGC TGG G	77.1
K44A_R	C CCA GCC ATA ATA ACC TGC CAG ACA CAG GGT ACC C	77.1
K44M_F	G GGT ACC CTG TGT CTG ATG GGT TAT TAT GGC TGG G	75.6
K44M_R	C CCA GCC ATA ATA ACC CAT CAG ACA CAG GGT ACC C	75.6
K44Q_F	G GGT ACC CTG TGT CTG CAA GGT TAT TAT GGC TGG G	75.9
K44Q_R	C CCA GCC ATA ATA ACC TTG CAG ACA CAG GGT ACC C	75.9
K44E_F	G GGT ACC CTG TGT CTG GAA GGT TAT TAT GGC TGG G	75.5
K44E_R	C CCA GCC ATA ATA ACC TTC CAG ACA CAG GGT ACC C	75.5
K44H_F	G GGT ACC CTG TGT CTG CAT GGT TAT TAT GGC TGG G	76.0
K44H_R	C CCA GCC ATA ATA ACC ATG CAG ACA CAG GGT ACC C	76.0

### Enzyme production and kinetic characterization

*Ec*FDH‐H enzymes carrying a C‐terminal His_6_‐tag were produced in a two‐step anoxic cultivation and purified by Ni‐ion affinity chromatography as described previously [15]. Briefly, *E. coli* cells were grown in stoppered 2‐L laboratory bottles containing 1 L lysogeny broth (LB) medium, including 0.4% (w/v) glucose, 15 mm formate, 10 μm Na_2_SeO_3_, and 1 mm Na_2_MoO_4_. Initially, bacterial cells were incubated for ~7 h at 37 °C after which *Ec*FDH‐H production was induced by addition of isopropyl β‐d‐1‐thiogalactopyranoside (IPTG, *c*_Final_ = 100 μm) and cultivation was continued for an additional 24 h at 30 °C. All the following steps, except centrifugations, were performed inside an anaerobic cabinet to preserve enzyme activity. Bacterial cells were resuspended in loading buffer (pH 7.5) containing 50 mm potassium phosphate, 150 mm sodium gluconate, 3 mm sodium azide, 2 mm dithiothreitol, and 40 mm imidazole and lysed by addition of lysozyme (*c*_Final_ = 0.1 g·mL^−1^) and sonication on ice. The cell lysate was clarified by consecutive centrifugation (30 000 ***g*** at 4 °C for 30 min) and filtration through a GD/X syringe filter (0.4 μm pore size; Whatman; Maidstone, UK). The clarified lysate was loaded onto a 5‐mL His‐trap HP column (Cytiva; Marlborough, MA, USA) that was equilibrated with loading buffer. Unbound lysate components were washed off from the column, and the target enzyme was eluted with loading buffer containing 300 mm imidazole. Imidazole was removed by a buffer exchange in PD‐10 desalting columns (Cytiva) using 25 mm MES buffer (pH 6.0) that was supplemented with 100 mm Na_2_SO_4_ to avoid enzyme precipitation caused by the decrease in the ionic strength of the enzyme solution upon imidazole removal. Enzyme solutions were concentrated by centrifugation using Vivaspin centrifugal concentrators (Sartorius; Göttingen, Germany), and their concentration was determined spectrometrically at *λ* = 280 nm using a NanoDrop DS‐11 spectrophotometer (DeNovix; Wilmington, DE, USA) and the molar extinction coefficient of *Ec*FDH‐H (115 × 10^3^ m ^−1^·cm^−1^) calculated using the Expasy ProtParam online tool (https://web.expasy.org/protparam/, [51]).

The formate oxidation activity of *Ec*FDH‐H enzymes was quantified spectroscopically at *λ* = 555 nm in a 200‐μL reaction volume contained in flat‐bottom 96‐well plates (Greiner Bio‐One; Kremsmünster, Austria) inside an anaerobic cabinet filled with a gas mixture of 5% H_2_ and 95% N_2_ using a Multiskan SkyHigh microplate spectrophotometer (Thermo Fisher Scientific; Waltham, MA, USA). Prior to enzyme addition, a reaction mix was prepared containing both, the native substrate sodium formate (10 mm) and the artificial substrate benzyl viologen (BV^2+^) dichloride (2 mm) dissolved in 50 mm phosphate buffer (pH 7.5). The formate oxidation reaction was initialized by addition of 5 μL enzyme solution containing variable amounts of *Ec*FDH‐H variants (WT: 1.5 μg; K44R: 7.5 μg; K44E, K44M, K44A, K44H and K44Q: 30 μg) dissolved in 25 mm MES buffer (pH 6.0) containing 100 mm Na_2_SO_4_. The BV^2+^ consumption was followed at *λ* = 555 nm for 60 s (ε_555 nm_ (BV^+^) = 12 mm ^−1^·cm^−1^) at 25 °C. Kinetic analysis of *Ec*FDH‐H (WT) and the variant K44R was performed at either a fixed formate concentration (10 mm) and varying BV^2+^ concentrations (0.125–20 mm) or a fixed BV^2+^ concentration (2 mm) and varying formate concentrations (0.125–200 mm) using the above‐mentioned formate oxidation assay. The apparent *K*_m_ values ($K_{m}^{\text{Formate}}$ and $K_{m}^{{\mathrm{BV}}^{2+}}$) were determined by nonlinear regression analysis of the substrate conversion data using the Michaelis–Menten model.

### Spectroscopic enzyme analysis

UV–Vis absorption spectra of *Ec*FDH‐H (WT) and K44 variants (2–3 mg·mL^−1^ enzyme dissolved in 25 mm MES buffer (pH 6.0) containing 100 mm Na_2_SO_4_) were recorded inside an anaerobic cabinet at *λ* = 250 nm–900 nm using a Multiskan SkyHigh spectrophotometer equipped with a μDrop Duo Plate (pathlength: 0.5 mm; Thermo Fisher Scientific). The UV–Vis absorbance signals at *λ* ~ 400 nm were quantified using the baseline subtraction function of the Peak Analyzer tool of the OriginPro software (Version 2023, OriginLab; Northampton, MA, USA) and *λ* = 300, 350, 450, 500, 550, and 600 nm as anchor points. CD spectra of *Ec*FDH‐H (WT) and obtained K44 variants (~0.5 mg·mL^−1^) dissolved in 25 mm MES buffer (100 mm Na_2_SO_4_, pH 6.0) were recorded under an anaerobic atmosphere. This was done by first transferring a 300‐μL sample into a QS‐110 quartz cuvette with a pathlength of 1 mm (Hellma; Müllheim, Germany) inside an anaerobic cabinet followed by hermetically sealing with a polytetrafluoroethylene stopper and spectroscopic analysis outside of the anaerobic cabinet using a Chirascan V100 CD spectrometer (Applied Photophysics; Leatherhead, UK) at 1.0 nm bandwidth, 0.5 nm step size, and 1.0 s measurement time per point with a protein‐free buffer as blank. Thermal unfolding experiments were performed at *T* = 25–55 °C with equilibration at each temperature for 60 s, and smoothing of spectra obtained from triplicate measurements using the Savitsky–Golay algorithm (8 points per window).

### Enzyme structure prediction & molecular dynamics simulation

Structural models of *Ec*FDH‐H variants were modeled using the MODELLER program (Discovery Studio 2019, BIOVIA; San Diego, CA, USA) with the published structure of the oxidized *Ec*FDH‐H (PDB ID: 1FDO) [21] as a template. Structural models with the lowest discrete optimized protein energy (DOPE) score (−10338.4) were selected as targets for molecular docking. Formate docking in the active site of *Ec*FDH‐H enzymes was performed using the CDOCKER program in Discovery Studio (default parameters) with a publicly available structure of the formate molecule (PubChem ID: 283, https://pubchem.ncbi.nlm.nih.gov/compound/283). The structures of the four predicted enzyme–substrate complexes were further optimized using the Protein Preparation Wizard software tool Jaguar of Maestro (version 7.6, Schrödinger; New York City, NY, USA) by energy minimization, that is the numerical determination of minima in their potential energy landscape starting from high energy conformations until the RMSD of the modeled structure was reduced below 0.5 Å. The residue p*K*_a_ values were estimated using the Prop*K*a online tool (https://www.ddl.unimi.it/vegaol/propka.htm [38]), with the modeled structures as input. The binding of formate in the *Ec*FDH‐H active site was analyzed by MD simulation using the Desmond MD engine (D. E. Shaw Research; New York City, NY, USA) of Maestro and the RMSD of selected regions in enzyme–ligand complexes was analyzed using the Simulation Interaction Diagram Wizard of the Maestro graphics user interface. The simulation system was built using a simple point charge (SPC) water model and a cubic box with an extended buffer dimension of 10 × 10 × 10 Å. In addition to the formate–*Ec*FDH‐H complex, the system contained Na^+^ counterions for neutralizing the charges in the simulation system. Pre‐equilibration was performed for 5 ns using the isothermal‐isobaric canonical ensemble at constant temperature, number of molecules, and pressure (NPT). MD simulations were performed using the OPLS4 all‐atom force field [52, 53] and NPT ensemble at a temperature of 318 K (45 °C) and standard pressure (1.01325 bar) for a 150 ns period at 150 ps/frame. All enzyme structures were visualized using the PyMOL Molecular Graphics System (Version 1.7.2; Schrödinger) in this study.

## Conflict of interest

The authors declare no conflict of interest.

## Author contributions

FLL: conceptualization; methodology; investigation; data curation; formal analysis; visualization; writing—original draft; writing—review and editing. ML: project administration; funding acquisition; supervision; conceptualization; formal analysis; visualization; writing—review and editing.

## Peer review

The peer review history for this article is available at https://www.webofscience.com/api/gateway/wos/peer‐review/10.1111/febs.70048.

## Data Availability

Additional data supporting the results of this study are available upon reasonable request.
